# COVID-19 and medical professionalism in a pandemic

**DOI:** 10.1136/postgradmedj-2020-138344

**Published:** 2020-10-10

**Authors:** Denis Harkin

**Affiliations:** Regional Vascular Surgery Unit, Royal Victoria Hospital, Belfast Health and Social Care Trust, Belfast, Northern Ireland BT12 6BA, UK

## EDITORIAL

The global pandemic caused by transmission of the novel severe acute respiratory syndrome coronavirus 2 (SARS-CoV-2) and resultantCOVID-19^1^ has created a crisis worldwide for health, healthcare and society. Doctors and healthcare workers will confront fears, and endure risks, making many difficult life-or-death decisions to treat patients and support colleagues as we confront this pandemic. United, in common purpose, we shall prevail against this generational challenge reliant upon our medical professionalism.

On 31 December 2019, the WHO China country office was notified of a severe contagious novel pneumonia outbreak in Wuhan, China, and concerned by the severity of illness and rapidity of spread, it was declared a Public Health Emergency of International Concern on 31 December 2019.^1^ The infection due to SARS-CoV-2 and the resultant coronavirus sisease first identified in 2019 was named COVID-19 by the WHO and declared a global pandemic on 11 March 2020.^1 2^ COVID-19 is asymptomatic or causes mild illness in most, but in a significant minority causes severe interstitial pneumonia and type-1 respiratory failure with florid systemic inflammatory response leading to multisystem organ failure and death. Worldwide, millions of people have been infected and hundreds of thousands have died, and among them are many front-line healthcare workers and surgeons.^1–4^ Epicentres have been overwhelmed by the demand for critical care support even in countries with well-resourced healthcare networks and have had to divert any available resource to cope with the pandemic surge.^1 2 5 6^ Our doctors will be asked to make many challenging decisions as this global pandemic now rages across Europe, the UK and Ireland.

Doctors will face unique challenges while managing the pandemic, including the personal risks of infection and the professional challenges of healthcare rationing, clinical priorities and working within a severely restricted health service.^7^ Doctors are also aware that significant collateral damage will arise from delays to diagnosis and treatment of other acute and chronic conditions. In practice, medical professionalism will involve the interaction between doctors and patients, and this should be a partnership based on respect, integrity and accountability.^8^ Prerequisite to a *healthy* patient–doctor relationship is trust; the patient must be able to place trust in their doctor to act in their best interests. Physicians in ancient times pledged upon the Oath of Hippocrates^3^ to act: ‘for the benefit of my patients, and abstain from whatever is deleterious or mischievous’. More recently, the American Board of Internal Medicine Foundation defined three fundamental principles of professionalism: the primacy of patient welfare, patient autonomy and social justice.^4^ In the UK, the Royal Society of Physicians defined medical professionalism as ‘a set of values, behaviours and relationships that underpins the trust the public has in doctors’.^9^ Upon these professional foundations are built the professional codes of both the UK’s General Medical Council^8^ and Irish Medical Council.^9^

Here we discuss how the key principles of medical professionalism, as set out by the American Board of Internal Medicine Foundation,^47–9^ may guide us as we strive to act in patients’ best interests and for the greater good of society during this greatest public health emergency for many generations.

## THE PRIMACY OF PATIENT WELFARE

Doctors have a primary responsibility to act in the best interests of their patients, without being influenced by any personal consideration.^7 8 10^ We provide care with compassion to vulnerable patients in extraordinary moments of fear, anxiety and doubt. Patients with COVID-19 can progress rapidly to severe type-1 respiratory failure, necessitating intubation, ventilation and critical care management.^1 2 5 6^ In the earliest epicentre of the outbreak in Wuhan, China, the death rate was as high as 5.25%.^2 5^ Worldwide deaths have mostly occurred in elderly patients and those with co-morbid disease. Epicentres, overwhelmed by demand, have had insufficient ventilators for all in clinical need and have directed finite and scarce resources to those who are most likely to survive.

Altruism is defined as the selfless concern for the well-being of others.^8^ COVID-19 has been transmitted within the hospital setting to infect healthcare workers, inpatients and visitors.^6^ Indeed, during the global pandemic, thousands of healthcare workers have died with COVID-19 succumbed to the disease. Our doctors, nurses and healthcare professionals, despite those risks, have continued to selflessly place themselves at risk to help patients and support each other. However, to sustainably care for others we must care for ourselves, and that demands we don effective personal protective equipment (PPE) and adhere to infection-control protocols even if that delays or reduces patient contact in an emergency.

In a pandemic, some individual patients’ best interests may come secondary to the primacy of societies greater good.^8^

## PATIENT AUTONOMY

In law, autonomy is often considered a negative right, rather a right to refuse treatment, sometimes termed non-interference.^8^ In contrast, to interpret autonomy positively would arguably entitle everyone to any requested treatment, regardless of medical advisability or competing claims for finite resources.^8 11 12^ However, to interpret autonomy in that positive respect would be considered incompatible with other ethical principles of non-maleficence (first do no harm), social justice (fair distribution of finite or scarce resources) and indeed with the practical reality of the provision of healthcare provision in a pandemic.^12^

In partnership with patients, the doctor has a duty to be honest, and educate and empower patients so they may make the appropriate informed choices about their medical care. During this pandemic, elderly patients and those with co-morbid disease may be considered most vulnerable, and yet are also the least likely to survive. We as doctors are not obligated to offer treatments that are considered to be futile. However, to withhold or withdraw precious life support from one individual considered less worthy for use in another more worthy patient creates a real dilemma for the doctor as patient advocate and public servant.^8^

The practice of medicine can be distinguished by the need for good judgement in the face of uncertainty. We must rely on our professionalism to do the right thing and be open and honest in communication with patients and families. If we are to maintain the public trust, we must also be candid when treatment choices are restricted by the availability of resources rather than the clinical needs.

In a pandemic, when finite resources become scarce, some patient’s choices will be restricted and in some situations withheld.

## SOCIAL JUSTICE

The principle of social justice in healthcare obliges us to take into consideration the needs of all patients and availability of resources as we appraise our individual patients’ needs.^4^ We have seen that in pandemic epicentres, highest death rates have coincided with a breakdown of overburdened local healthcare systems. The critical care demand has overwhelmed even well-resourced healthcare systems such that subjective evaluations of the benefits and burdens of life support have had to direct finite resources to those patients most likely to survive.^5 6 12^ Grave decisions such as these should not be taken alone but by working in partnership with patients, colleagues and teams. Barriers to multidisciplinary team (MDT) decision-making created by the pandemic can be ameliorated by senior front-line clinical leadership, the use of ‘hot’ (bedside) and ‘virtual’ (videoconferencing) MDTs, and by deferring those most difficult decisions on prioritisation and healthcare rationing to ethical review panels composed of both medical and lay experts. Social justice also dictates we must consider the indirect harm that will occur from delays to diagnosis, treatment, procedures and surgeries. As the pandemic surge dampens, we must advocate for the safe reintroduction of urgent and then routine care to minimise that collateral damage.

To combat this pandemic, we need best guidance on the therapeutic strategies for COVID-19 and how best to protect ourselves and our patients.^6^ Reassuringly, collaborative efforts of academics and professional associations have rapidly and effectively disseminated best evidence and expert consensus to guide clinicians. Our employers also have a legal and ethical responsibility to protect staff and they must ensure they have sufficient PPE and training to minimise the risks of in-hospital transmission.^6^ Doctors will have to balance many competing interests, such as personal risk, best interests, society’s interests, clinical priorities, ceilings-of-care, withholding and withdrawing care.^12^ They will also need to adapt to modified service delivery, intense work-patterns, moratoriums on annual and study leave, and altered career-pathways. In exceptional circumstance, staff and students will be asked to act outside of their normal role but should strive to work within their competence with rapid retraining, upskilling and supervision.

Justice dictates that as doctors will endure these changes, society (including employers and our professional bodies) should ensure staff are appropriately supported through and beyond the pandemic in respect to health, well-being, career, indemnity, licencing and revalidation.

## CONCLUSIONS

Doctors have a duty and responsibility to act according to the best values of medical professionalism, and society has a corresponding duty to ensure the infrastructure and support available allow doctors to deliver those responsibilities as safely as possible. Our professionalism will help guide doctors to do the right thing and strive to get the best available outcomes for their patients during this COVID-19 pandemic. There will of course be pain, but we shall persevere and together hope to build a better future to honour those who are lost along the way.
